# Experts’ Opinions on the Sustainable Use of Digital Health Tools for Effective Future Pandemic Preparedness and Response: Questionnaire Study

**DOI:** 10.2196/84164

**Published:** 2026-06-16

**Authors:** Kabelo Leonard Mauco, John H Holmes, Anthony Luberti, Badisa Mosesane

**Affiliations:** 1 Center for Global Health Perelman School of Medicine University of Pennsylvania Philadelphia, PA United States; 2 Department of Biostatistics, Epidemiology, and Informatics Perelman School of Medicine University of Pennsylvania Philadelphia, PA United States; 3 Department of Pediatrics Perelman School of Medicine University of Pennsylvania Philadelphia, PA United States; 4 Department of Biomedical and Health Informatics Children’s Hospital of Philadelphia Philadelphia, PA United States; 5 Global Health Informatics Department of Biomedical and Health Informatics Children’s Hospital of Philadelphia Philadelphia, PA United States

**Keywords:** digital health, eHealth, public health informatics, digital epidemiology, pandemic preparedness, pandemic response, digital health readiness, global health, COVID-19, artificial intelligence, AI

## Abstract

**Background:**

The COVID-19 pandemic demonstrated the potential role of digital health tools in enhancing pandemic preparedness and response. These tools became essential, supporting not only health care delivery but also decision-making, communication, case identification, contact tracing, surveillance, vaccination rollout, and intervention evaluation. The interest in applying digital health tools to pandemic preparedness and response motivated conversations about digital epidemiology—a field of study that aims to provide insight into health and disease determinants by leveraging diverse digital data sources. In a globalized world, effective preparedness and response to pandemics require coordinated global action.

**Objective:**

This study investigates experts’ opinions on strategies for improving global health security through the effective use of digital epidemiology, considering the current landscape of digital determinants of health.

**Methods:**

Epidemiologists, public health specialists, data scientists, and professionals with expertise in various components of digital health were recruited through convenience and snowball sampling methods. Their opinions were elicited using an electronic questionnaire developed by the authors in Research Electronic Data Capture (REDCap; Vanderbilt University). To ensure a global perspective, participants were recruited from Africa, North America, Oceania, and Europe. Thematic analysis and the strengths, weaknesses, opportunities, and threats (SWOT) analysis framework were used to analyze participants’ responses.

**Results:**

Most participants were familiar with the concept of digital epidemiology and expressed positive sentiments about its potential in strengthening global health security. Privacy and security, along with ethical and legal considerations, were ranked by most experts as high priority areas that decision-makers and implementers must consider to ensure sustainable integration of digital epidemiology tools in future pandemic preparedness and response. A SWOT analysis of participants’ views on the promise of digital epidemiology revealed fewer strengths and more weaknesses compared to other components of the analysis framework.

**Conclusions:**

This study highlights the growing recognition of digital epidemiology as a critical tool for enhancing global health security, particularly using nontraditional data sources and emerging technologies, including artificial intelligence. The study affirms the need for a globally coordinated approach to governance, regulation, and investment in digital health infrastructure to ensure the responsible and effective application of digital innovations in epidemiological practice.

## Introduction

The COVID-19 pandemic underscored the critical role of digital health tools in enhancing pandemic preparedness and response. It is estimated that the COVID-19 pandemic caused more than 2 million deaths worldwide [1]. A report of the Independent Panel for Pandemic Preparedness and Response concluded that the alert system was too slow, the World Health Organization (WHO) was underresourced, and global political leadership was absent [2]. The study by de Souza et al [3] identified 5 critical areas associated with the global failure in facing the pandemic: deficiency in the global alert system and the fragility of the international health regulations, problems of the international response to the pandemic related to global health governance, the dispersed global adoption of the elimination strategy (zero-COVID), fragile control of the disease with a narrow reading of the associated problems, and finally, global setbacks in achieving the sustainable development goals. The need for coordinated global efforts and effective early detection and timely surveillance for a robust pandemic and epidemic early warning and preparedness has been widely discussed amid the COVID-19 pandemic [4].

Digital health tools became a necessity and their popularity increased during the pandemic not only for the provision of health care services but also to support decision-making, communication and information, case identification, contact tracing, surveillance and monitoring, and rolling out of vaccination programs, as well as evaluation of interventions [5-7]. Digital health technologies illustrated the potential to support diverse needs and approaches for early warning and forecasting of epidemics [8]. Nevertheless, in their report, Al Knawy et al [9] acknowledge that COVID-19 highlighted widespread chronic underinvestment in digital health that hampered public health responses to the pandemic. The often ad hoc adoption and use of digital health during the pandemic further illuminated its potential to transform future pandemic preparedness and response efforts [10].

The discussion on applying digital health tools toward future pandemic preparedness and response has motivated frequent conversations around digital epidemiology. Digital epidemiology has been defined as a field of study that aims to provide insight into health and disease determinants in human populations by building on diverse digital data sources [11]. Furthermore, the study by Tarkoma et al [11] posits that digital epidemiology is envisaged as having the potential to contribute toward global health security through inputs, such as syndromic surveillance, public health surveillance, and early pandemic detection.

In the era of globalization, efficient preparedness and response to future pandemics requires a united global action [12,13]. However, realizing the full potential of digital epidemiology as part of such global efforts becomes elusive due to persistent geopolitical tensions as well as the global digital divide and digital access gap [14]. This study aims to investigate experts’ opinions on possible means of improving global health security through the effective use of digital epidemiology within the context of the current global landscape of digital determinants of health.

## Methods

### Sampling and Recruitment of Study Participants

Epidemiologists, public health specialists, data scientists, and professionals with expertise in the various components of digital health were recruited using convenience sampling. To gather a global perspective, potential study participants were recruited from Africa, North America, Oceania, and Europe.

For recruitment of potential participants from Africa, an initial email was sent to the general membership of the pan-African health informatics association called Health Informatics in Africa. For the United States, initial recruitment of study participants involved sending an email to members of the American Medical Informatics Association. Snowball sampling method was also used. The initial email to potential study participants described the study objectives and the role of a potential study participant. Study data were collected and managed using the Research Electronic Data Capture (REDCap; Vanderbilt University) tool hosted at the Children’s Hospital of Philadelphia [15]. The email package also included a link to an electronic questionnaire developed using the REDCap platform. The electronic questionnaire included a consent form that a potential study participant had to agree to before proceeding to answer the questionnaire.

Recruitment of potential study participants was also done at the Medical Informatics Europe 2023 conference held in Göteborg, Sweden. Paper flyers about the study were placed at convenient places throughout the conference venue. In addition to containing details about the study objectives and participant’s role, the paper flyers also included a QR code with a link to the electronic questionnaire developed using REDCap.

Recruitment of study participants continued until data saturation was reached, defined for this study as the point at which additional data no longer generated new insights. As an electronic questionnaire was used for this study, the authors aimed to comply with the Checklist for Reporting Results of Internet E-Surveys [16]. The consolidated criteria for reporting qualitative research were followed for reporting of this study. The completed checklist is provided in Multimedia Appendix 1.

### Study Questionnaire Design and Development

The initial draft of the study questionnaire was designed based on a review of relevant literature. Following an iterative process, the authors developed a study questionnaire aimed at capturing participants’ opinions on effective approaches to improving global health security using digital epidemiology within the context of the current global landscape of digital determinants of health.

A panel consisting of 5 staff members from the Department of Biomedical and Health Informatics at the Children’s Hospital of Philadelphia and the Perelman School of Medicine at the University of Pennsylvania was consulted to ensure content validity of the study questionnaire. The panel received the initial draft questionnaire via email and could make suggestions for improvement where needed. Their suggestions were discussed and agreed upon among the authors and final updates were made on the questionnaire. The structured questionnaire included both closed-ended (Likert-scale responses) and open-ended questions, allowing for analysis of trends as well as insights into experts’ recommendations. This approach facilitated a comprehensive evaluation of participants’ responses.

### Data Analysis

Thematic analysis was used to analyze the qualitative responses from the questionnaire, following an iterative process [17]. All textual data were imported into NVivo software (version 12; Lumivero) to facilitate systematic coding and theme identification. Initial codes were generated based on both predefined study objectives and emerging patterns from the data. Through repeated review and refinement by all authors, broader themes and subthemes were developed to capture key insights. Consensus on the final themes was reached through discussion among all authors.

Descriptive statistics were computed to summarize responses. Participants’ responses were analyzed by applying the strengths, weaknesses, opportunities, and threats (SWOT) analysis framework [18] to the digital epidemiology approach. The responses were independently reviewed by 2 coauthors (KLM and BM) and assigned to one of the SWOT categories. Any disagreements or discrepancies in the assignment of responses to SWOT categories were discussed, and consensus on the final categorization of each response was reached by the authors.

### Ethical Considerations

This study involved seeking experts’ opinion through the use of an electronic questionnaire—what is commonly classified as a minimal risk study, in which the probability and magnitude of harm or discomfort arising from participation were not greater, in and of themselves, than those ordinarily encountered in daily life or during the performance of routine physical or psychological examination or tests. Nevertheless, ethics approval for the study was obtained from the institutional review board at the Children’s Hospital of Philadelphia (institutional review board protocol number: 22-020667). Participants received the survey electronically using REDCap, and consent to participate was obtained from all participants through a consent form embedded in the electronic questionnaire (REDCap). Only participants who provided consent were able to proceed and complete the questionnaire. Participants’ privacy and confidentiality were ensured throughout the study. REDCap is a secure, web-based platform designed to support electronic data capture for research and operational projects, and it is compliant with the Health Insurance Portability and Accountability Act and the General Data Protection Regulation. The study did not collect any personally identifiable information. All data collected were anonymized and reported in aggregate form only. Participants did not receive any compensation for taking part in the study.

## Results

Following the data collection phase (February 2023-February 2024), a total of 22 fully completed questionnaires were retained for analysis, while incomplete responses were excluded. Questionnaires were deemed incomplete if participants failed to respond to any of the mandatory sections or to questions measuring key outcome variables. The study participants represented diverse geographical regions, including Africa (7/22, 32%), the Americas (7/22, 32%), Europe (6/22, 27%), and Oceania (2/22, 9%). A detailed breakdown of this distribution is presented in Table 1.

**Table 1:** Characteristics of study participants (N=22) and countries represented in a global expert survey on digital epidemiology conducted from February 2023 to February 2024.

Region, subregion, and country				Participants, n (%)
**Africa (n=7)**				
	**Eastern Africa**			
		Rwanda	1 (4.5)	
		Uganda	2 (9.1)	
		Malawi	1 (4.5)	
	**Western Africa**			
		Ghana	1 (4.5)	
	**Central** **Africa**			
		Cameroon	1 (4.5)	
	**Southern Africa**			
		South Africa	1 (4.5)	
**Americas (n=7)**				
	**North America**			
		United States	6 (27.3)	
		Canada	1 (4.5)	
**Europe (n=6)**				
	**Western Europe**			
		Switzerland	3 (13.6)	
		France	1 (4.5)	
		Netherlands	1 (4.5)	
	**Southern Europe**			
		Greece	1 (4.5)	
**Oceania (n=2)**				
	**Australia and New Zealand**			
		Australia	2 (9.1)	

The study participants represented diverse professional backgrounds, with 14 (62%) participants identifying as informaticians, 4 (19%) participants as public health specialists, 3 (14%) participants as medical doctors, and 1 (5%) as a philosopher (Table 2). Participants were required to identify with only 1 mutually exclusive professional category.

**Table 2:** Occupation distribution of study participants (N=22) in a global expert survey on digital epidemiology conducted from February 2023 to February 2024.

Occupation	Participants, n (%)
Public health	4 (19)
Medical doctor	3 (14)
Informatician	14 (62)
Philosopher	1 (5)

This study aimed to explore experts’ opinions on what they would consider as optimal conditions necessary for the use of digital epidemiology to enhance future pandemic preparedness and response. Accordingly, it was essential to assess participants’ awareness of the concept of digital epidemiology, as this could influence the study’s findings and interpretations. As illustrated in Table 3, most study participants were aware of the concept of digital epidemiology, with more than half of the participants either strongly agreeing or agreeing that they were familiar with digital epidemiology.

**Table 3:** Awareness of the concept of digital epidemiology among study participants (N=22) in a global expert survey conducted from February 2023 to February 2024.

Response category	Participants, n (%)
Strongly agree	11 (50)
Agree	8 (35)
Neutral	1 (5)
Disagree	1 (5)
Strongly disagree	1 (5)

Participants who either strongly agreed or agreed (n=19 participants) that they were aware of the concept of digital epidemiology (Table 3), generally expressed positive sentiments regarding its potential impact and effectiveness in improving global health security (Table 4).

**Table 4:** Study participants’ sentiments regarding the potential impact and effectiveness of digital epidemiology (n=19) in a global expert survey conducted from February 2023 to February 2024.

Statement	Strongly agree, n (%)	Agree, n (%)	Neutral, n (%)	Disagree, n (%)	Strongly disagree, n (%)
Digital data can enhance epidemiology purposes	13 (68)	4 (21)	2 (11)	0 (0)	0 (0)
Digital epidemiology can be effectively applied at a global level toward a coordinated global health surveillance	11 (57)	6 (32)	2 (11)	0 (0)	0 (0)
Digital epidemiology is reliable	4 (21)	12 (63)	2 (11)	1 (5)	0 (0)

**Figure 1:**
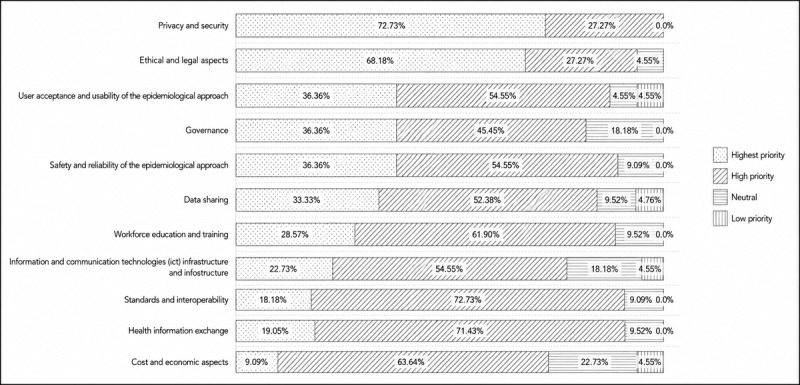
Priority ranking of key readiness indicators for effective integration of digital epidemiology in pandemic preparedness and response, as reported by study participants (n=19) in a global expert survey conducted from February 2023 to February 2024.

Effective integration of digital epidemiology as part of a united global action toward future pandemic preparedness and response would require a certain level of readiness from the global community [7,18]. Key readiness indicators for decision-makers and implementers to consider are outlined in Figure 1. Study participants ranked these indicators based on their perceived importance and priority. In order of importance and priority, from highest to lowest, the indicators were ranked as follows: privacy and security, ethical and legal aspects, user acceptance and usability of the epidemiological approach, governance, safety and reliability of the epidemiological approach, data sharing, workforce education and training, information and communications technology infrastructure and infostructure, standards and interoperability, health information exchange, and costs and economic aspects (Figure 1). Participants also identified associated strengths, weaknesses, opportunities, and threats. It is noteworthy that fewer strengths and more weaknesses were identified relative to the other components of the SWOT analysis (Table 5).

**Table 5:** Strengths, weaknesses, opportunities, and threats (SWOT) analysis of opinions on the effective integration of digital epidemiology in pandemic preparedness and response (n=19) in a global survey of experts conducted from February 2023 to February 2024.

SWOT analysis categories	Themes emerging from participants’ responses
Strengths	Increasing investments in digital technologies by countries Existence of digital health strategies by countries
Weaknesses	Limited competent workforce Weak digital health infrastructure Digital divide Poor data quality Limited data sharing Digital illiteracy Limited use of artificial intelligence or machine learning Weak ethical and legal considerations Disconnect between public health departments and hospitals
Opportunities	Global open standards that promote data exchange Global design International coordination on ethical considerations Inclusion of the nonhealth sector
Threats	Data security Global economic challenges Patient acceptance Ethics Political interference Political unawareness

## Discussion

### Principal Findings

This qualitative study gathered insights from 22 experts (Table 1). Although the sample size may be considered relatively small, it is appropriate for the exploratory nature of the research and emphasizes achieving information power [19]. This approach provides a rich, in-depth, context-specific preliminary evidence rather than statistical generalization. In qualitative research designed to identify patterns across data, recruitment of between 15 and 30 participants has been commonly found to be adequate [20].

Digital epidemiology is a relatively new field that has grown rapidly in recent years, with its humble beginnings traced back to when researchers began mining the increasing amount of internet data for epidemiological purposes [21]. The growth of the field, and thus increased awareness, has been fueled by the growing utility of new data sources and artificial intelligence (AI) in epidemiology [22]. The ad hoc use of digital epidemiology methodologies and techniques during the COVID-19 pandemic likely contributed to the heightened awareness of the field [23]. This may explain why most study participants, especially in light of their professional backgrounds, reported being aware of the concept of digital epidemiology (Table 2). However, it is important to note that the attitude of stakeholders, rather than their awareness, may play a more crucial role in driving sustainable use of digital epidemiology. The technology acceptance model features prominently among the methodologies used to measure attitudes toward technology adoption [24,25]. According to the technology acceptance model, perceived usefulness (PU) is characterized by an individual’s belief that using technology improves their job performance, while perceived ease of use (PEOU) refers to their belief that using technology requires minimal effort [24]. Both PU and PEOU are primary drivers that influence attitudes toward using technology [25]. Most study participants agreed that digital data can enhance epidemiological purposes (PU), that digital epidemiology can be effectively applied at a global level toward coordinated global health surveillance (PU), and that digital epidemiology is reliable (PU+PEOU). This is illustrated in Table 4 and reflects participants’ positive attitudes toward digital epidemiology, particularly regarding its potential impact and effectiveness in improving global health security.

The Riyadh Declaration on digital health is a global call to action promoting the development of infrastructure and best practices necessary for sharing high-quality, real-time data both locally and globally in the fight against pandemics. Its goal is to enable health systems to access actionable information that can inform timely responses to public health threats [26]. Similarly, the US Centers for Disease Control and Prevention launched the Data Modernization Initiative to enhance the quality, availability, and use of data related to pandemics and public health emergencies [27]. Together, these frameworks serve to guide the integration of digital innovations into national and global health systems as part of coordinated efforts to strengthen preparedness and response to future pandemics. A shared theme emphasized across these frameworks is that leveraging digital epidemiology to address global health threats requires a more robust and integrated global health architecture that is capable of better preparing local, national, regional, and global public health partners to predict, prevent, detect, assess, and respond effectively to emerging threats. This calls for intentional efforts by governments and public health authorities to mitigate digital exclusion at all levels (global, regional, national, and local) and to address data governance and interoperability challenges inherent in using and sharing diverse data sources [28].

Privacy and security, along with ethical and legal considerations, were ranked by most experts as high priority areas that must be considered by decision-makers and implementers to ensure sustainable integration of digital epidemiology tools in preparedness and response to future pandemics (Figure 1). Digital epidemiology, as previously explained, makes effective use of data not originally intended for epidemiological purposes. Examples of such data include internet searches, social media posts, webpage access logs, mobile phone network data, mobile health apps, wearable devices, sensor generated data, call center records, health and patient registries, and environmental monitoring data [21,23]. As some of these data are generated outside traditional public health systems and those involved in generating such data may not have consented to or even be aware of their use for epidemiological purposes, indeed this raises legitimate concerns related to potential data privacy breaches, security vulnerabilities, and ethico-legal issues. The pertinent literature suggests that data on one’s health status have the potential to be reused by third parties to discriminate and restrict individual rights, and that institutional and government access to personal data (eg, geospatial data) could inhibit the exercise of basic freedoms, if individuals feel watched as to what they do or with whom they spend time [29]. In the context of the existing global digital divide and digital access gap, reliance on digital epidemiology to drive pandemic preparedness and response strategies may inadvertently perpetuate existing health disparities, benefiting overrepresented populations in the data, while not providing gains to and even possibly harming populations whose data are underrepresented [30]. In light of the current fragmented regulatory landscape and in the absence of a common set of principles on data privacy, security, and ethics, a global coordinated approach is required to establish frameworks that will ensure safe and responsible use of digital epidemiology as part of a unified global effort toward future pandemic preparedness and response [31]. Findings from a study based on the unified theory of acceptance and use of technology framework indicated that cost did not moderate behavioral intention to adopt the technology [32]. This may help explain why, in this study, experts ranked cost and economic aspects lower among the key readiness factors for the adoption of digital epidemiology (Figure 1).

The WHO’s Global Strategy on Digital Health 2020 to 2025 provides a structured road map to guide member states in systematically integrating digital health technologies into their national health systems. According to WHO, the vision of the global strategy is to improve health for everyone, everywhere by accelerating the development and adoption of appropriate, accessible, affordable, scalable, and sustainable person-centric digital health solutions to prevent, detect, and respond to epidemics and pandemics [33]. Existence of such a document is indeed a strength in creating an enabling environment for the use of digital epidemiology in future pandemic preparedness and response. Increasing investments in digital technologies by countries and existence of digital health strategies by countries (Table 5) are part of the strategic objectives advanced by the document. The 4 strategic objectives outlined in the Global Strategy on Digital Health are as follows: (1) promote global collaboration and advance the transfer of knowledge on digital health, (2) advance the implementation of national digital health strategies, (3) strengthen governance for digital health at global, regional, and national levels, and (4) advocate people-centered health systems that are enabled by digital health [32]. These strategic objectives align with concerns highlighted as weaknesses by experts in Table 5. In addition to data privacy, security, and ethics already discussed, experts identified sociopolitical challenges and economic constraints as potential threats to a global approach to digital epidemiology (Table 5). This aligns with the study by Davis [34], which discusses the political determinants of digital health, defining them as the intersecting areas of national and international law and policy, local and global governance, civic engagement, and the influence of transnational commercial interests. The author explains that these factors collectively shape access to, uptake of, and the design of digital health systems [34]. As a case point, global geopolitical tensions and varying digital health maturity levels of nations can significantly impact data sharing in public health, resulting in lapses in global health security.

The full potential of digital epidemiology in achieving coordinated pandemic preparedness and response can indeed be leveraged through advancements in global design and international collaborations on digital health–related issues (Table 5). Opportunities exist in open health data standards [35], which promote interoperability and enable effective data sharing across diverse settings. The WHO’s extension of the Global Strategy on Digital Health 2025 to 2027 endeavors to provide critical guidance on AI in health, including the ethics and governance of AI for health, and aims to support member states through global workshops on ethical AI implementation [36] and the code of ethics for health information professionals of the International Medical Informatics Association [37], are but examples of opportunities that offer a pathway toward safe and responsible use of digital epidemiology as part of a unified global effort for future pandemic preparedness and response. As previously explained, digital epidemiology uses data sources beyond the traditional health sector to gain insight into disease patterns and trends. As such, a significant opportunity exists in the continued efforts by global health actors to engage representation from nonhealth sectors in plans for future pandemic preparedness and response (Table 5). These sentiments are emphasized in the WHO Pandemic Agreement which sets out principles, approaches, and tools for better international coordination across a range of areas to strengthen the global health architecture for pandemic prevention, preparedness, and response [38]. The WHO Pandemic Agreement perhaps holds great promise toward significantly contributing to the creation of an enabling environment for the effective role of digital epidemiology in future pandemic preparedness and response efforts.

### Limitations

This study is subject to limitations inherent in its design. The use of convenience and purposive sampling methods limits the generalizability of the findings. The sample may have excluded critical perspectives from underrepresented geographic regions or professional backgrounds. For instance, within the North American region, participants from the United States were heavily overrepresented, with only 1 participant from Canada, thereby excluding perspectives from other countries in the region. The composition of professional backgrounds represented in the sample may have influenced participant responses, potentially skewing perceptions toward more favorable views of digital epidemiology. These notable imbalances may have constrained the diversity and balance of expert opinions captured.

### Conclusions

This study explored experts’ perspectives on the potential, readiness factors, and challenges associated with integrating digital epidemiology into global pandemic preparedness and response strategies. The findings underscore a growing recognition of digital epidemiology as a critical tool for enhancing global health security, particularly using nontraditional data sources and emerging technologies such as AI. Participants demonstrated high awareness and positive attitudes toward the utility and applicability of digital epidemiology, especially in advancing coordinated global health surveillance. However, the study also identified key barriers that must be addressed to ensure sustainable integration of digital epidemiology into health systems. These barriers include concerns related to data privacy, security, ethics, sociopolitical influences, and digital inequities across and within countries. The study affirms the need for a globally coordinated approach to governance, regulation, and investment in digital health infrastructure. By highlighting the complex interplay between technological, institutional, political, and social determinants of digital epidemiology, this research contributes important insights into how global health actors can leverage digital innovations responsibly and effectively for epidemiological purposes. Future research directions should focus on developing and validating context-specific frameworks that offer clear guidance for the responsible and sustainable integration of digital epidemiology into existing health systems, to support preparedness for and response to future pandemics.

## Data Availability

The data used and analyzed during this study are available from the corresponding author upon reasonable request.

## Appendix

Multimedia Appendix 1 COREQ checklist.

## Abbreviations

AI: artificial intelligence
PEOU: perceived ease of use
PU: perceived usefulness
SWOT: strengths, weaknesses, opportunities, and threats
WHO: World Health Organization
