# Supplementary material for: Experts’ Opinions on the Sustainable Use of Digital Health Tools for Effective Future Pandemic Preparedness and Response: Questionnaire Study
Source: JMIR Public Health Surveill. 2026 Jun 16;12:e84164. doi: 10.2196/84164 (PMC13319999; doi:10.2196/84164)
Supplement: Multimedia Appendix 1 [file publichealth_v12i1e84164_app1.pdf]

Consolidated criteria for reporting qualitative studies (COREQ): 32-item checklist

No. Item	Guide questions/description	Notes/Comments
**Domain 1: Research team and reflexivity**		
Personal Characteristics		
1. Interviewer/facilitator	Which author/s conducted the interview or focus group?	Reported on Page 21
2. Credentials	What were the researcher's credentials? E.g. PhD, MD	Reported on Page 1
3. Occupation	What was their occupation at the time of the study?	Fulbright Scholar
4. Gender	Was the researcher male or female?	Male
5. Experience and training	What experience or training did the researcher have?	PhD in Telehealth, with 7 years experience in qualitative and quantitative research methods
Relationship with participants		
6. Relationship established	Was a relationship established prior to study commencement?	Reported on pages 4-5
7. Participant knowledge of the interviewer	What did the participants know about the researcher? e.g. personal goals, reasons for doing the research	Reported on pages 4-5
8. Interviewer characteristics	What characteristics were reported about the interviewer/facilitator? e.g. Bias, assumptions, reasons and interests in the research topic	Reported on pages 4-5

**Domain 2: study design**		
Theoretical framework		
9. Methodological orientation and Theory	What methodological orientation was stated to underpin the study? e.g. grounded theory, discourse analysis, ethnography, phenomenology, content analysis	Reported on pages 6-7
Participant selection		
10. Sampling	How were participants selected? e.g. purposive, convenience, consecutive, snowball	Reported on pages 4-5
11. Method of approach	How were participants approached? e.g. face-to-face, telephone, mail, email	Reported on page 5
12. Sample size	How many participants were in the study?	Reported on page 8
13. Non-participation	How many people refused to participate or dropped out? Reasons?	N/A
Setting		
14. Setting of data collection	Where was the data collected? e.g. home, clinic, workplace	Reported on page 5
15. Presence of non-participants	Was anyone else present besides the participants and researchers?	N/A
16. Description of sample	What are the important characteristics of the sample? e.g. demographic data, date	Reported on pages 9-10
Data collection		
17. Interview guide	Were questions, prompts, guides provided by the authors? Was it pilot tested?	Reported on page 6
18. Repeat interviews	Were repeat interviews carried out? If yes, how many?	N/A
19. Audio/visual recording	Did the research use audio or visual recording to collect the data?	N/A
20. Field notes	Were field notes made during and/or after the interview or focus group?	N/A
21. Duration	What was the duration of the interviews or focus group?	About 15 minutes
22. Data saturation	Was data saturation discussed?	Yes, Reported on page 5
23. Transcripts returned	Were transcripts returned to participants for comment and/or correction?	N/A
**Domain 3: analysis and findings**		

Data analysis		
24. Number of data coders	How many data coders coded the data?	Reported on pages 6-7
25. Description of the coding tree	Did authors provide a description of the coding tree?	Reported on pages 6-7
26. Derivation of themes	Were themes identified in advance or derived from the data?	Reported on pages 6-7
27. Software	What software, if applicable, was used to manage the data?	NVivo 12, and<br><br>Research Electronic<br><br>Data Capture<br><br>(REDCap)
28. Participant checking	Did participants provide feedback on the findings?	N/A
Reporting		
29. Quotations presented	Were participant quotations presented to illustrate the themes/findings? Was each quotation identified? e.g. participant number	No
30. Data and findings consistent	Was there consistency between the data presented and the findings?	Reported on pages 8-20
31. Clarity of major themes	Were major themes clearly presented in the findings?	Reported on pages 12-14
32. Clarity of minor themes	Is there a description of diverse cases or discussion of minor themes?	Reported on pages 11-14
